# Evidence-based mapping of design heterogeneity prior to meta-analysis: a systematic review and evidence synthesis

**DOI:** 10.1186/2046-4053-3-80

**Published:** 2014-07-23

**Authors:** Michelle D Althuis, Douglas L Weed, Cara L Frankenfeld

**Affiliations:** 1 EpiContext, Washington, DC 20003, USA; 2 DLW Consulting Services, LLC, Salt Lake City, UT 84103, USA; 3 Department of Global and Community Health, George Mason University, 4400 University Drive, MS 5B7, Fairfax, VA 22030, USA

**Keywords:** Heterogeneity, Evidence map, Systematic review, Meta-analysis, Sugar-sweetened beverages, Type 2 diabetes

## Abstract

**Background:**

Assessment of design heterogeneity conducted prior to meta-analysis is infrequently reported; it is often presented post hoc to explain statistical heterogeneity. However, design heterogeneity determines the mix of included studies and how they are analyzed in a meta-analysis, which in turn can importantly influence the results. The goal of this work is to introduce ways to improve the assessment and reporting of design heterogeneity prior to statistical summarization of epidemiologic studies.

**Methods:**

In this paper, we use an assessment of sugar-sweetened beverages (SSB) and type 2 diabetes (T2D) as an example to show how a technique called ‘evidence mapping’ can be used to organize studies and evaluate design heterogeneity prior to meta-analysis.. Employing a systematic and reproducible approach, we evaluated the following elements across 11 selected cohort studies: variation in definitions of SSB, T2D, and co-variables, design features and population characteristics associated with specific definitions of SSB, and diversity in modeling strategies.

**Results:**

Evidence mapping strategies effectively organized complex data and clearly depicted design heterogeneity. For example, across 11 studies of SSB and T2D, 7 measured diet only once (with 7 to 16 years of disease follow-up), 5 included primarily low SSB consumers, and 3 defined the study variable (SSB) as consumption of either sugar or artificially-sweetened beverages. This exercise also identified diversity in analysis strategies, such as adjustment for 11 to 17 co-variables and a large degree of fluctuation in SSB-T2D risk estimates depending on variables selected for multivariable models (2 to 95% change in the risk estimate from the age-adjusted model).

**Conclusions:**

Meta-analysis seeks to understand heterogeneity in addition to computing a summary risk estimate. This strategy effectively documents design heterogeneity, thus improving the practice of meta-analysis by aiding in: 1) protocol and analysis planning, 2) transparent reporting of differences in study designs, and 3) interpretation of pooled estimates. We recommend expanding the practice of meta-analysis reporting to include a table that summarizes design heterogeneity. This would provide readers with more evidence to interpret the summary risk estimates.

## Background

Meta-analyses, which are quantitative methods for pooling results from epidemiologic studies, inform research priorities and health policy. Combining similar studies asking a similar research question is fundamental to the interpretability of summary risk estimates [1]. Combining results in a meta-analysis from studies that are designed to answer different scientific questions may lead to imprecise and possibly invalid inferences [2,3].

An assessment of the similarity of studies (that is, design heterogeneity) is a fundamental element of a meta-analysis of epidemiological studies [3-8]. There are two major types of heterogeneity: statistical heterogeneity and design heterogeneity (sometimes referred to as clinical and methodological diversity) [9]. Statistical heterogeneity is purely a mathematical assessment; evidence of statistical heterogeneity indicates that there is greater statistical variance between the study results than would be expected by chance if the effect size was similar across studies [8,10]. Design heterogeneity, in contrast, involves the extent to which the studies being considered for inclusion in a meta-analysis differ in study design, including population studied, specificity of exposure measurement, uniformity of diagnostic criteria (in the outcome), confounders measured, concomitant exposures measured, and statistical models [3,7].

Reviews of the practice of meta-analysis in observational epidemiology have observed that investigators often emphasize the summarization function over the assessment of heterogeneity [2,11]. Additionally, in a systematic overview of meta-analyses, we found fewer than a third of 47 eligible meta-analyses of lifestyle and dietary risk factors for type 2 diabetes (T2D) reported a detailed characterization of design heterogeneity that was used to guide the quantitative pooling of study results (manuscript in preparation). In contrast, more than 90% of the meta-analyses reported some assessment of statistical heterogeneity (Q statistic or I^2^ index). These observations illustrate that the assessment of design heterogeneity frequently occurs after statistical heterogeneity has been identified. In practice, design heterogeneity assessment would be informative if undertaken before any quantitative summarization takes place [2].

In 2013, two journals focusing on research synthesis methods (Systematic Reviews and Research Synthesis Methods) emphasized the importance of qualitative evaluation of studies selected for meta-analysis, calling for more strategies to aid conduct and reporting [12,13]. In this paper, we present a strategy for objectively and transparently characterizing design heterogeneity of epidemiologic studies prior to meta-analysis.

## Methods

Evidence-based mapping was used as a tool to diagram and tabulate data across a group of studies selected for meta-analysis, with the following three primary objectives:

1. to document differences in exposure (intervention), comparator, outcome, and study design and population characteristics;

2. to assess the design features and population characteristics associated with specific definitions of the exposure (intervention), comparator and outcome; and

3. to evaluate the diversity in modeling strategies (for example, assessment of confounding for observational epidemiology studies) and suggest simple summary measures to benchmark susceptibility of the exposure risk estimate to the influences of included (and excluded) co-variables in multivariable regression models.

We sought to summarize the detailed work of multiple evidence maps created to meet these objectives into a single table with a universal adaptable format. The aim of this table is to facilitate the reporting of design heterogeneity, which is fundamental to developing a protocol, analyzing data, and interpreting meta-analyses.

### Tools

Evidence maps are a relatively new tool used to transparently generate a clear visual depiction of complicated data, either in the form of a diagram or a table [14]. Evidence maps have been used to set research priorities by displaying existing research landscapes without linking study designs to study results [14-23]. Precisely because evidence mapping seeks to organize studies without summarizing results, they are natural tools for assessing design heterogeneity prior to meta-analysis. Therefore, we expanded evidence mapping methods by demonstrating their usefulness in planning a meta-analysis. This work is guided by previously published evidence maps whose focus was research priority setting [14-23] and the existing standards for conducting and reporting of systematic reviews of observational research [4,24]. Evidence maps were created in Excel (Microsoft, Redmond, Washington, USA); however, it is possible to conduct the work using other database software.

### Definition of design heterogeneity

In this paper, design heterogeneity refers to diversity across studies in sociodemographic and health characteristics of the populations studied; methods of study execution and data ascertainment; exposure (intervention), comparator and outcome definitions; and statistical approaches, as well as analyses conducted and reported.

### Evidence-based mapping framework: steps for evaluating design heterogeneity

In order to present the evidence-based mapping framework in a way that other investigators can easily translate to their own research questions, the next section describes each step generally. The details of the application of the framework to a specific example are described in the subsequent section, including the systematic search process. This framework is designed to be dynamic. Although we recommend completing this work prior to finalizing a protocol and analysis plan for a meta-analysis, updating will be necessary when new data become available. We recommend that at least three investigators thoroughly use this framework: an author to abstract data at the onset and another to verify accuracy and a consensus of experts to review completed maps and evaluate aspects of design heterogeneity that may importantly influence meta-analysis of the selected studies.

Prior to applying this three-step framework, a PICO (participants, intervention (or exposure), comparator, and outcome) table is completed to identify key research components and to develop/clarify the research question [25]. Once a group of studies have been selected for a meta-analysis, diversity is assessed across all included studies for each of the four PICO elements. An evaluation of confounding is added when including observational studies in the analysis. This framework can be used to document design heterogeneity across many study types, including randomized clinical trials.

#### Step 1: To assess diversity across selected studies for each participants, exposure/intervention, comparator, and outcome element

The goal of the first step of this framework is to evaluate the extent of diversity within each of the four PICO elements, although not in the same order. The framework begins with an assessment of the exposure variable (or intervention) across selected studies for two reasons: the exposure definition often is the driver of the analysis, and it is usually documented in the most detail in a publication.

For each study, the definition used for exposure is abstracted, including information on the measurement tools, timing of variable collection, and method/criteria (self-report, interviewer administered, medical or biochemical test). When possible, the exact language used to ascertain exposure status or details of the test performed is recorded. A diagram (evidence map) is created to describe how definitions of the study variable related to one another, quantitatively and qualitatively. The description of the variable definition, using the original language from the publication as much as possible, is summarized in a text box. Text boxes are organized to group together exposure variables with similar definitions. Similar definitions are physically grouped together in the diagram, and the review investigator assigns descriptive ‘category headings’ accordingly. The category of the exposure variable most frequently employed across studies is placed at the top of the diagram, with other categories arranged in order of decreasing frequency. An evaluation of whether the most frequently used definition is indeed the most appropriate definition was not undertaken at this point in the review process. The step should be examined later along with study quality and risk of bias.

The resulting map visually depicts patterns within the exposure definitions and is used to preliminarily evaluate whether the collective group of studies directly address the review question or address more than one distinct question. It also facilitates an initial assessment of frequently occurring subgroups of the exposure variable, which could be considered for stratified or sensitivity analyses in a meta-analysis.

In an etiologic example, the comparator is often the lowest exposure category and therefore included as part of the evaluation of the study variable. In analyses of randomized trials or nonrandomized studies, step 1 is repeated in order to evaluate diversity among definitions of the comparator across selected studies.

Step 1 is also repeated for the outcome variable (with particular attention to diagnostic method/criteria) and as needed for variables describing the study characteristics and population, examples include study location/ethnicity, gender, study size, study duration, timing of participant assessments, and baseline population characteristics such as age, body size, or health status. Univariate statistics (n, median, proportion, range) were used to describe the diversity of variable definitions (exposure, outcome, and co-variables) across included studies.

#### Step 2: To assess the design features and population characteristics associated with specific definitions of the exposure

The second step is to assess whether specific definitions of the exposure variable tended to aggregate with specific study design features or population characteristics. Using separate diagrams for each category of the exposure variable (identified in Step 1), important design features and population characteristics are listed for each study and qualitatively inspected to identify emerging patterns. Particular attention is focused on differences between categories of the exposure variable that are identified in Step 1 as potentially not directly answering the review question. Likewise, among exposure categories from studies directly answering the review question, the aggregation of study design/population characteristics is used to augment decisions from Step 1 about stratified/sensitivity analyses in a future review/meta-analysis.

Step 2 can be repeated as necessary to understand whether certain comparators or outcomes are associated with design or population characteristics.

#### Step 3: To evaluate the diversity in multivariable modeling strategies and assessment of confounding

The aim of step 3 is to evaluate co-variables selected for models by primary studies and to facilitate the selection of an adequately adjusted model or models for combining by meta-analysis of observational studies. Evidence-based mapping is used to visually display the patterns of co-variables adjusted for in each model as reported by each publication. A table summarizes how the exposure variable is analyzed in each study (for example, continuous measure or categories) and tallies the number of models from each publication and the number of covariates adjusted for in each model.

Every regression model is listed in sequential order as presented in the original research publication and a checklist format is used to summarize covariates adjusted for in each model. Covariates most frequently adjusted for in multivariable models across all studies are listed in the map header. A check denotes inclusion of a covariate in the model and a superscript is added to indicate the timing of the measurement of the covariate (for example, BL for baseline). Less frequently adjusted covariates are listed in a single column of the table.

For each multivariable model, the percent change in the exposure-outcome risk estimate from the age-adjusted model is calculated using the following equation:

(age-adjusted relative risk - multivariable adjusted relative risk)/(age-adjusted relative risk - 1).

This provides a quantitative assessment of the degree of fluctuation in the exposure-response risk estimate from the age-adjusted value, depending on the covariates included in a model.

Frequency (n), median, proportion, and range are used to describe across included studies the diversity in definitions of the study variable used for analysis (for example, how categories of exposure were defined), number of multivariable models presented, number of covariates adjusted for in multivariable models, and change in exposure-outcome risk estimate from the age-adjusted risk estimate.

#### Summarization of evidence-based mapping efforts

A single table, organized with sections for each PICO element, captures important findings and bridges the more practical need to concisely document and report design heterogeneity. We adopted a format that would be flexible for summarizing the large amounts of complex data organized by evidence maps. Using cohorts as the unit of analysis, for each major category of exposure (as determined by Step 1), the following were summarized: the distribution of important design and population characteristics (as determined in Step 2: n, percent), operationalization of the study variable in the multivariable model (Step 3: n, percent), the number of multivariable models presented in the original publication (Step 3: median, range), the number of covariates in multivariable models (Step 3: median, range), and the change in exposure-outcome risk estimate from the age-adjusted risk estimate (Step 3: median, range).

### Illustration of method using prospective observational studies of sugar-sweetened beverages and type 2 diabetes

We illustrate the utility of an evidenced-based mapping framework using an example from nutritional epidemiology: sugar-sweetened beverages (SSB) and type 2 diabetes mellitus (T2D). This example is ideal for illustrating this framework, because studies of this relationship characteristically have considerable variability in study design.

#### Selection criteria

First, we identified published work for the example. We used an electronic search strategy to identify all cohort studies of dietary sugar intake and T2D. Published research that met the following inclusion criteria were identified for full text review: 1) a prospective observational study (that is, dietary sugar consumption was measured in chronologic time prior to measurement of T2D) and 2) a study analyzing the risk of T2D associated with dietary sugar intake, dietary patterns, or glycemic load/index. To address the possibility that electronic search strategies might omit publications of findings not important enough (for example, null findings) for inclusion in the title, keywords, or abstract, our search ascertained published research on dietary patterns as well as dietary sugar intake. Additionally, we identified reviews and meta-analyses of epidemiologic studies on this topic in order to examine their reference lists.

#### Systematic search

We conducted database searches of PubMed and Scopus (inception to 10 March 2014). We limited our search of the PubMed database to human studies and English language publications, and used the following combination of search terms and medical subject headings (inception to 19 September 2013: 2,005 titles): sweetening agents, energy intake, calories, caloric intake, fructose, glucose, sucrose, monosaccharides, disaccharides, dietary carbohydrates, soda, sugar beverage, sweetened beverage, soft drink, dietary sugar, juice, sugar intake, sugary foods, sweets, sweet foods, carbohydrate intake, glycemic index, glycemic load, macronutrients AND diet, dietary patterns, dietary intake AND cohort studies, incidence, follow-up, prospective studies, meta-analysis AND Diabetes Mellitus, type 2 diabetes). We conducted a similar title, abstract, and keyword search of the Scopus database (1,143 titles): (diet* and sugar*) OR (diet* and pattern*) OR soda OR juice OR (sweet* and drink*) OR (sweet* and beverage*) OR (sweet* and food*) and (‘type 2 diabetes’). The search results were downloaded into Refworks (©Proquest 2012). Titles, abstracts, and keywords of all articles were examined, and those that continued to meet the inclusion criteria were ascertained for further full text review.

To ensure accurate identification of eligible studies, we conducted two pilot tests of our methodology prior to implementing the search described above. First, we assessed and revised a search strategy after retrieval and review of citations from several years, 2010 to 2013. The revised search strategy included more terms and more specific terms for dietary sugar, glycemic load/index and energy intake. This led to a broader, more inclusive search and the review of more titles. Second, two authors independently reviewed a subset of citations identified by our search strategy for eligibility (titles from 2012). Because both authors identified the same articles (inter-rater reliability = 100%), decisions regarding inclusion/exclusion reliably were based on a review by one author.

#### Identification and tracking of eligible publications

**Figure 1:**
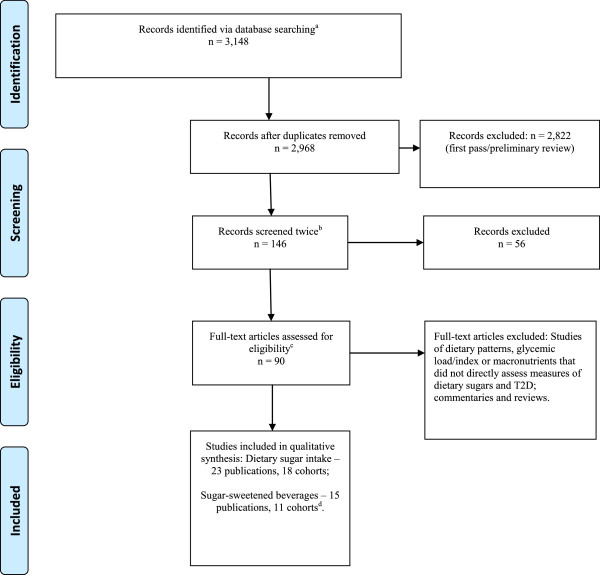
**Systematic search for eligible studies of dietary sugar intake and type 2 diabetes. (a)** 2,005 from PubMed and 1,143 from Scopus data base searches. **(b)** Titles remotely on topic were screened twice. **(c) **We completed a full-text review of all studies of dietary patterns, glycemic load/index, and carbohydrates to assess whether a measure of dietary sugar was examined individually. We also reviewed the full text and bibliographies of studies of sugar-sweetened beverages (SSB), juices, sugars, macronutrients and key reviews and commentary. **(d) **We identified three cohorts with multiple publications, from which we selected for this synthesis the one publication in which SSB was either the main study variable or the definition was the clearest. We identified two publications of the Health Professionals Follow-up study (HPFS); of these two publications, the one that assessed SSB as the primary study variable was selected for inclusion [34] and the other that presented analyses stratified by the main variable, caffeine consumption, was excluded [35]. We selected one of the three publications from the Nurse’s Health Study (NHS). Bazzano and coworkers [39] reported risk separately for a one-increment serving of sugar-sweetened colas, fruit punch, low calorie cola, and other carbonated beverage. In a personal communication from a 2010 meta-analysis [50], Malik and coworkers report a risk estimate for SSB intake, but the definition was not provided nor was the analysis adjusted for age. Although not ideal, the Bhupathiraju *et al*. analysis of SSB, stratified by caffeinated and caffeine-free beverage consumption, provides a clear definition (sugar-sweetened carbonated beverages) and analysis, and therefore was selected for inclusion in this paper [35]. Our final exclusion was a 2013 publication of EPIC-France [31], from which all participants were represented by an included EPIC publication [29].

A flow chart tracked eligible publications identified by the literature searches and illustrated a two-stage evaluation process (Figure 1).

As part of the process of identifying eligible cohorts we displayed how epidemiologic studies of SSB fit into the broader field of research on dietary sugar intake and T2D. We tabulated the cohorts that published on measures of dietary sugar intake (including SSB) by study size. The number of publications and corresponding cohorts were depicted for each definition of dietary sugar intake, including sweetened beverages and macronutrients (sucrose, fructose, and glucose). Table 1 was based on the World Health Organization and the Food and Agricultural Organization of the United Nations definitions [26] of dietary sugar intake as ‘all monosaccharides and disaccharides added to foods by the manufacturer, cook, or consumer; sugars naturally present in honey, syrups and fruit juices [27].’ Table 1 facilitated identifying the studies that focus on SSB as a subset of all studies on dietary sugar intake.

**Table 1:** Publications and cohorts that report the relationship between measures of dietary sugar intake and type 2 diabetes

		**Macronutrients**				
**Cohorts (reference)**	**Sugar-sweetened beverages (SSB) - broadly defined**^**a**^	**Total sugars**^**b**^	**Sucrose**	**Fructose**	**Glucose**	**Fructose and glucose**
>*25*,*000 Participants*						
BWHS [28]	√					
EPIC-All [29,30]	√^(29)^	√^(30)^				
EPIC-FR [31]	√					
EPIC-NL [32]		√				
EPIC-P [33]			√	√	√	
HPFS [34,35]	√					
IWHS [36]			√	√	√	
JPHC [37]	√					
MelC [38]		√				
NHS [35,39,40]	√^(35,39)^		√^(40)^			
NHSII [41]	√					
SCHS [42]	√					
WHS [43]		√	√	√	√	
*10*,*000 TO 24*,*999 Participants*						
ARIC [44]	√					
*5000 to 9*,*999 Participants*						
MESA [45]	√					
*1000 to 4*,*999 Participants*						
EPIC-Nor [46]		√	√	√	√	
FMC [47]	√	√	√	√	√	√
Jfact [48]	√					
Total publications:	15	6	6	5	5	1
Total unique cohorts represented^c^	11	4	6	5	5	1
	9 Publications: 8 Cohorts					

^a^SSB was broadly defined to include studies that defined sweetened beverages as either SSB only or as soft drinks (either sugar or artificially sweetened).

^b^Total sugars = disaccharides and monosaccharides.

^c^Total cohorts represented enumerates unique cohorts. Eight of 10 countries are represented in EPIC-All, which overlaps with country specific EPIC publications except for Norway and Greece.

ARIC, Atherosclerosis Risk in Communities Study; WHS, Women’s Health Study (B, Black, I, Iowa); EPIC-All, P, N, NL, FR, European Prospective Investigation of Cancer (InterAct Study, Potsdam, Norfolk, Netherlands, France); FMC, Finnish Mobile Clinic Health Examination Survey; HPFS, Health Professional’s Follow up Study; Jfact, Study of Japanese factory workers; JPHC, Japan Public Health Center-based Prospective Study; MESA, Multi-ethnic Study of Atherosclerosis; NHS, Nurse’s Health Study; SCHS, Singapore Chinese Study.

#### Data abstraction

For eligible prospective observational studies of SSB and T2D, we created detailed data abstraction tables. For each study, we abstracted data on sample size and population characteristics (for example, country, baseline age and body size), SSB definition and consumption at baseline, T2D diagnosis, dietary assessment timing and tools, duration of follow-up, timing of ascertainment of beverage consumption, variables included in multivariable models, and statistical analyses. We present this work without linking study design features to study results. We recommend this step in order to minimize as much as possible selection bias when planning a protocol for a subsequent meta-analysis. One author abstracted data at the onset, all authors contributed to strategy and map designs, and another author verified the accuracy of data abstracted at the end stage.

#### Participants, exposure/intervention, comparator, and outcome elements

The following PICO elements were specified for this work:

1. Participants/study design: adults from the general population without T2D/prospective observational studies.

2. Exposure: sugar-sweetened beverage consumption (our example is etiologic; therefore the ‘I’ in PICO is exposure).

3. Comparator: low or no consumption of sugar-sweetened beverages.

4. Outcome: incident T2D.

#### Step 1: Assessing diversity across selected studies for each participants, exposure/intervention, comparator, and outcome element

**Figure 2:**
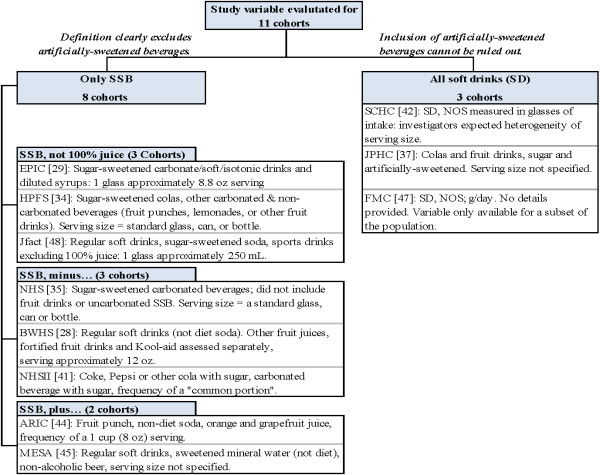
**Step 1: Categorizing cohorts according to the definition of the study variable, sugar-sweetened beverages.** ARIC, Atherosclerosis Risk in Communities Study; BWHS, Black Women's Health Study; EPIC, European Prospective Investigation of Cancer (InterAct Study); FMC, Finnish Mobile Clinic Heath Examination Survey; HPFS, Health Professional's Follow up Study; Jfact, Study of Japanese factory workers; JPHC, Japan Public Health Centre-based Prospective Study; MESA, Multi-Ethnic Study of Atherosclerosis; NHS, Nurse's Health Study; SCHS, Singapore Chinese Health Study; SD, soft drink; SSB, sugar-sweetened beverage.

Evidence maps were used to categorize studies based on the definition of the exposure variable, outcome, and population characteristics. Exposure characterization took into account the type of beverage, data collection instruments, and frequency/timing of data collection (Figure 2). Variation in definitions of T2D was evaluated based on criteria for diagnosis and method of ascertainment such as by a physician or self-report. Using the study as the unit of analysis, univariate statistics (n, median, proportion, range) were used to describe across included cohorts heterogeneity of SSB intake (exposure and comparator), T2D diagnosis (outcome), and the following population/study characteristics: study location, gender, study size, duration of follow-up, baseline BMI, and baseline SSB consumption.

#### Step 2. Describing design features and population characteristics associated with the exposure across eligible cohorts

**Figure 3:**
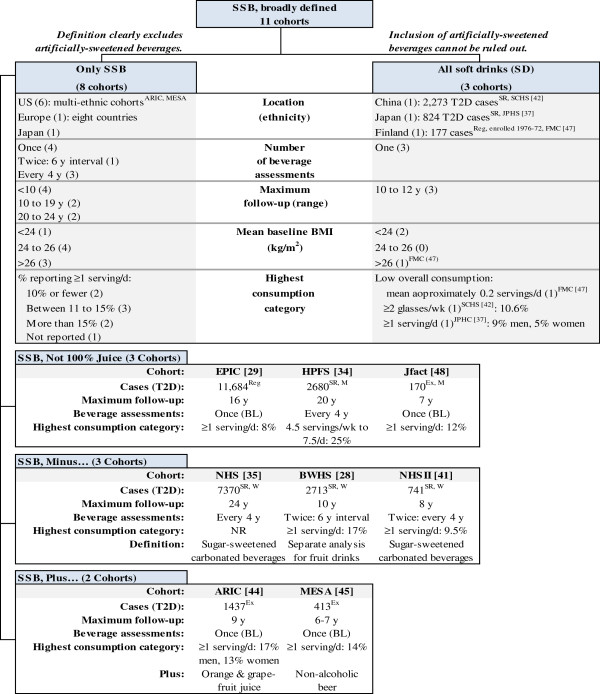
**(Step 2). Sweetened beverage definitions by cohort description and methods: studies of incident type 2 diabetes (T2S).** T2D diagnosed by self-report of symptoms/medication or physician diagnosis (SR); linkage to a registry (Reg); or upon exam (Ex). NR, not reported; BL, baseline; M, men; W, women; ARIC, Atherosclerosis Risk in Communities Study; BWHS, Black Women's Health Study; EPIC, European Prospective Investigation of Cancer (InterAct Study); FMC, Finnish Mobile Clinic Heath Examination Survey; HPFS, Health Professional's Follow up Study; Jfact, study of Japanese factory workers; JPHC, Japan Public Health Centre-based Prospective Study; MESA, Multi-Ethnic Study of Atherosclerosis; NHS, Nurse's Health Study; SCHS, Singapore Chinese Health Study; SD, soft drink; SSB, sugar-sweetened beverage.

Cohorts were organized in a diagram according to category of the sweetened beverage consumption identified in Step 1. Design and population characteristics for cohorts falling into each beverage category were summarized. This provided an organized illustration of whether specific definitions of SSB tended to aggregate with specific study design features or population characteristics (Figure 3).

#### Step 3. Describing modeling strategies across eligible cohorts (assessing confounding)

**Figure 4:**
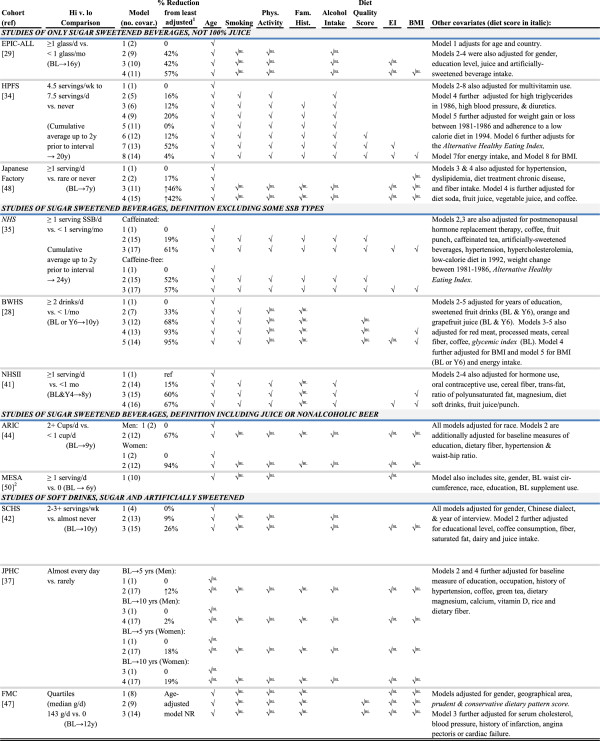
**(Step 3). Covariates adjusted for in multivariable models of sugar-sweetened beverages and type 2 diabetes: 11 cohorts.** 1. Calculated as proportion change from the age-adjusted model or a fairly simple model: (RRage adjusted - RRmodel)/(RRage adjusted - 1). ↑ denotes an increase in the risk estimate. 2. For the MESA cohort, the model information was based on author correspondence reported in a 2010 meta-analysis [50]. BL, adjustment variable based on baseline assessment. All cohorts used Cox proportional hazards models, except JPHC [37], which used logistic regression.

Evidence-based mapping visually displayed the patterns of covariates adjusted for in each model of SSB and T2D as reported by each publication (Figure 4). A check denoted adjustment for covariates age, smoking, physical activity, family history, alcohol intake, diet quality score, energy intake, and body mass index. Overall and within important strata of the study variable, diversity of multivariable modeling strategies was described by summarizing operationalization of the SSB intake (n, percent), the maximum number of models of SSB and T2D presented in the original publication (median, range), the maximum number of covariates in models (median, range), and the maximum change in SSB-T2D risk estimate from the age-adjusted risk estimate (median, range).

#### Summarization of evidence-based mapping efforts (Table 2)

**Table 2:** **Design heterogeneity across 11 cohorts assessing risk of type 2 diabetes**, **stratified by inclusion of artificially sweetened beverages in the study variable definition**

	**Only sugar-sweetened beverages (SSB) (eight cohorts)**		**Soft drinks (SD) (three cohorts)**	
**Study design and population and characteristics**	**N**	**%**	**n**	**%**
Study location				
United States	6	75%	0	
Europe	1	13%	1	33%
Japan	1	13%	2	67%
Gender				
Women	3	38%	0	
Men	2	25%	0	
Both men and women	3	38%	3	100
Case of T2D				
1 to 4,999	2	25%	1	33%
500 to 4,999	4	50%	2	67%
5,000+	2	25%	0	
Duration of follow-up				
<10 years	4	50%	0	
10 to 14 years	1	13%	3	100%
15+ years	3	38%	0	
Mean baseline body mass index (kg/m2)				
<24	1	13%	2	67%
24 to 26	4	50%	0	
>26	3	38%	1	33%
Number/timing of beverage assessment				
Once at baseline (study length range from 7 to 16 years)	4	50%	3	100%
Twice (6-year interval)	1	13%	0	
Every 4 years	3	38%	0	
Proportion of study participants reporting ≥ serving/day				
10% or fewer or low consumption	2	25%	3	100%
Between 11 and 15%	3	38%	0	
More than 15%	2	25%	0	
Not reported	1	13%	0	
Method of type 2 diabetes (T2D) diagnosis				
Self report with validation	4	50%	2	67%
Direct measurement/medical records	4	50%	1	33%
**Operation of study variable in multivariable models**: **highest versus lowest category of consumption**				
Highest consumption category:				
2+ drinks or cups/day^ARIC,BWHS^	2	25%	0	33%
1+ glasses or servings/day	6	75%	1	67%
<1 serving/day	0		2	
Lowest consumption category:				
Never	2	25%	1	33%
never or rarely	5	63%	2	67%
<1 cup/day^ARIC^	1	13%	0	
**Characterization of multivariable models**	**Range**	**Median**	**Range**	**Median**
No multivariable models presented	1 to 8	4	2 to 4	3
Maximum number of co-variables in multivariable models^a^	9 to 17	14	14 to 17	15
Maximum% change in SSB-T2D risk from age-adjusted estimate	46 to 95%	61%	2 to 26%	18%

^a^Covariates most frequently adjusted for in multivariable models of the 11 eligible cohorts include physical activity (11 of 11), smoking (11), energy intake (11), BMI (10), family history (9), alcohol intake (8), education (5), and diet quality score (4).

Using cohorts as the unit of analysis, for categories defined by SSB-intake (as determined by Step 1), the following was tabulated: the diversity of design and population characteristics, the study variable and the outcome (as determined in Steps 1 and 2: n, percent), operationalization of the SSB in the multivariable model (Step 3: n, percent), the number of multivariable models of SSB-T2D presented in the original publication (Step 3: median, range), the number of covariates in SSB-T2D multivariable models (Step 3: median, range), and the change in SSB-T2D risk estimate from the age-adjusted risk estimate (Step 3: median, range).

## Results

### Literature search and identification of eligible publications

The search results are summarized in Figure 1. Briefly, a total of 3,148 titles were reviewed (2,005 from Pubmed and 1,143 from Scopus). After duplicate removal (N = 180), 2,968 titles were examined and reviewed in a two-step process. We identified 146 titles broadly on topic; a second review revealed that 90 were prospective epidemiology studies, commentary and reviews of sugar and T2D. Excluded were publications whose focus was not dietary sugar intake and T2D and those that were case–control, cross-sectional and ecologic studies (N = 56). Full-text review of the 90 publications identified 22 primary research publications of the relationship between dietary sugar intake and T2D [28-49] and one meta-analysis with previously unpublished data [50]. A bibliography search of systematic reviews and meta-analyses did not identify any more potentially eligible titles [50-52].

Table 1 summarizes 21 publications from 17 cohorts that report the relationship between the following measures of dietary sugar intake and T2D [28-48]: category of SSB (broadly defined including some studies which include both artificially and sugar-sweetened beverages) and sugar-related macronutrients (total sugars, sucrose, fructose, and glucose). Studies of SSB (broadly defined) represent the majority of the published work on measures of dietary sugar intake, with 15 publications from 11 cohorts, most with more than 5,000 study participants. Nine publications from eight cohorts analyze sugar-related macronutrients and T2D, with total sugars and sucrose being the most frequently assessed. With the exception of a Finnish study initiated prior to 1970, all studies of macronutrients have >25,000 participants. A very small Swedish study assessing cake and biscuit consumption [49] was not summarized by Table 1.

### Organizing and evaluating design heterogeneity among cohorts assessing sugar-sweetened beverages and type 2 diabetes

For the assessment of design heterogeneity, we selected one publication from each cohort that had multiple publications (n = 3 cohorts). We selected the one in which SSB was either the main study variable or the definition was the clearest. Details of unselected publications are noted at the bottom of Figure 1.

#### Step 1. Assessing diversity across selected studies for each participants, exposure/intervention, comparator, and outcome element

Study variables and outcomes were categorized into logical groups by definitions reported in each of the 11 eligible cohorts. No two cohorts define the main study variable alike. As shown in Figure 2, two broad definitions of sweetened beverage consumption emerged: 1) three studies used the nonspecific definition soft drinks (SD) that included both sugar and artificially-sweetened beverages, and 2) eight studies restricted the definition to SSB only. Three distinct subgroups were identified among cohorts defining the study variable as exclusively sugar-sweetened. The general definition ‘SSB, not 100% juice’ includes all drinks with added sugar (sodas, colas, other carbonated SSB, and noncarbonated SSBs such as fruit punches, lemonades or other fruit drinks). Two other SSB patterns were identified within the remaining five cohorts based on whether they excluded beverages (SSB minus, three cohorts) or included additional beverages (SSB plus, two cohorts) from the anchor definition (SSB, not 100% juice). We found that investigators more frequently excluded beverages from the anchor definition, most broadly noncarbonated soft drinks as an entire group or fruit drinks. Two studies added beverages to the definition, one orange and grapefruit juice and the other non-alcoholic beer. This detailed characterization of the study variable identified two broad research questions addressed by this series of selected studies: T2D risk associated with intake of 1) SSB only or 2) any SD (artificially or sugar-sweetened).

Of the 11 cohorts, method of diagnosis was based on self-report (n = 6; 3 of the 6 were studies of health professionals), registry linkage (n = 2), and an examination by a health professional (n = 3).

Univariate analysis of design features and population characteristics across the 11 cohorts revealed heterogeneity in study location (US: n = 6, Europe: n = 2, China/Japan: n = 3), size (>5000 cases: n = 2, 500 to 4999 cases: n = 6, 1 to 499 cases: n = 3), duration of follow-up (<10 years: n = 4, 10 to 14 years: n = 4, 15 or more years: n = 3); mean baseline body mass index (<24 kg/m^2^: n = 3, 24 to 26 kg/m^2^: n = 4, > 26 kg/m^2^: n = 4), ascertainment of diet (food frequency questionnaire: n = 8, diet history: n = 3), and frequency of diet assessment (baseline only: n = 7, twice every 6 years: n = 1, every 4 years: n = 3). We also found a relatively low consumption of SSB across the 11 cohorts with nearly half (n = 5) reporting that 10% or fewer participants consumed one or more servings per day.

#### Step 2. Describing design features and population characteristics associated with the study variable across eligible cohorts

The upper portion of Figure 3 compares design features and population characteristics of cohorts defining sweetened beverage consumption as SSB only and SD. SSB cohorts were mainly US-based (n = 6), completed diet assessments at least twice (n = 4), followed subjects for fewer than 10 years (n = 4), and reported a mean body mass index (BMI) ≥24 kg/m^2^ (n = 7). We identified three levels of SSB consumption ascertained at the baseline visit of these eight combined cohorts: frequency of one or more servings a day (highest consumption group for each study) was reported by 10% or fewer (n = 2), between 11 and 15% (n = 3) and more than 15% (n = 2) of cohort participants.

Design and population characteristics for SD cohorts presented differently. Two of three SD cohorts were Asian populations; the one western population was a small Finnish study that enrolled participants between 1967 and 1972. Mean BMI was <24 kg/m^2^ in two of the three studies, SD consumption was measured only at the baseline visit (10 to 12 years prior to maximum follow-up duration), and SD consumption overall for the three cohorts was low. Comparison of the study design features of cohorts assessing SSB only with SD suggests it would not be sensible to combine all eleven studies in a meta-analysis; instead the main pooled analysis should include the eight SSB-only studies.Further division of SSB cohorts into categories of SSB, SSB minus, and SSB plus uncovered patterns according to study design, as shown in the lower portion of Figure 3. Large studies of women (Black Women’s Health Study (BWHS), Nurse’s Health Study (NHS), NHSII) with multiple dietary assessments more narrowly defined SSB consumption as excluding noncarbonated drinks (SSB, minus). The multicultural cohorts initiated to study atherosclerosis (Multi-Ethnic Study of Atherosclerosis (MESA), Atherosclerosis Risk in Community study (ARIC)) more broadly defined SSB by including either juice or non-alcoholic beer. These cohorts have higher baseline SSB consumption when compared to studies defining SSB more narrowly. No clear pattern emerged for cohorts defining the study variable as SSB, not 100% juice. Stratification of a meta-analysis on SSB subcategories and gender may additionally be important for understanding pooled T2D risk estimates.

We used a similar process to evaluate design heterogeneity of the outcome definitions used in these cohorts. While several different criteria for T2D were used in the 11 cohorts, the main defining characteristic was whether the diagnosis was based on self-report (all included a validation study), physical examination, or linkage to a registry or other health database. With the exception of the European Investigation of Cancer (EPIC) study, which verified cases via a registry, the larger studies (>25,000 participants) relied on self-reported diagnoses. Three studies conducted routine physical exams.

#### Step 3. Diversity of modeling strategies (confounding)

Multivariable models compared risk in the highest category of consumption (quartile or quintile) to the lowest. Figure 4 summarizes the different definitions of high and low categories of sweetened beverage consumption. Among studies of SSB only, the highest consumption category was 1+ glasses or servings each day in 6 (of 8) cohorts and 2+ drinks or cups each day for 2 (serving sizes varied). In comparison, the highest consumption category for two of three cohorts evaluating SD was less than one serving per day. Never or rare sweetened beverage consumption was the most frequently employed reference group (7 of 11), followed by never consumption (3 of 11). ARIC was the only study to include more frequent consumption in the reference group: up to one cup of SSB per day.Figure 4 visually depicts multivariable models and the pattern of covariate adjustment across 11 cohorts. The majority of cohorts present a multivariate model adjusting for age, physical activity, smoking, family history, alcohol intake, energy intake and BMI. Four studies adjust for a diet quality score, although all measure and adjust for some aspect of diet. Many models further adjust for multiple other covariates (up to 17).

Many models use different definitions to adjust for the listed co-variables and 9 of 11 adjust for covariates as measured at baseline. For example, measures of other dietary factors ranged from one variable measuring dietary fiber to healthy eating scores based on the entire diet (for 3 cohorts only). Likewise body mass index is adjusted in many ways: as a continuous variable (3 of 11 cohorts), a categorical variable (5 of 11 cohorts), and as measured at baseline (6 of 11 cohorts).Multivariable models adjust for between 5 and 17 covariates which corresponded to a 46% to 95% maximum reduction from the age-adjusted model in T2D risk associated with SSB-only intake (Figure 4). Change in the risk estimate was most pronounced among the large cohorts of US women using sugar-sweetened carbonated beverages as the study variable: reductions were as large as 95% in the BWHS, 61% in the NHS, and 67% in the NHSII. Change in risk estimates with addition of covariates was less pronounced among SD cohorts (range, 2-26%).

#### Summarization of evidence-based mapping efforts

Table 2 concisely summarizes the considerable amount of variability in study design, population characteristics, and statistical analysis among the 11 cohorts of sweetened beverages and T2D. This table represents a proto-type for a universal table on study design heterogeneity summarizing key design features uncovered by detailed evidence-based mapping efforts and organized according to PICO elements. The results in Table 2 are presented stratified by SSB only and SD to display the association of different definitions of the study variable with specific design and population characteristics. In addition, Table 2 highlights diversity in statistical analysis and provides a benchmark for the potential for confounding overall and for the two primary definitions of sweetened beverage consumption.

## Discussion

Evidence-based mapping can be used as a tool to improve the assessment and reporting of design heterogeneity prior to meta-analysis of epidemiologic studies. The framework described herein is useful for all study designs, but particularly for observational epidemiologic studies, which are complex and rich in important detail. If studies are found to be similar enough to combine via meta-analysis, this framework is useful for evaluating diversity in study designs, particularly statistical methods; facilitating the logical categorization of studies for stratified and sensitivity analyses when designing a protocol or analysis plan for a meta-analysis; and developing tools for model selection in meta-analysis of observational studies reporting multiple multivariable models. A standard table for summarizing the results from the 3 steps in this framework is essential for displaying the multi-dimensionality of diversity across a group of selected studies and to aid interpretation of a pooled risk estimate.

Evidence maps are ideal tools for characterizing heterogeneity prior to a meta-analysis. Previously they have been used for research priority setting by the Cochrane Collaboration [53] and other organizations such as the Agency for Healthcare Research and Quality [54-56]. In addition to organizing a complex body of research, another defining feature of evidence mapping is that the mapping of study characteristics is undertaken without linking to study results [15]. Although we used prospective observational studies of SSB and T2D to explain our approach, the framework is robust and this strategy can be applied to other exposure-disease relationships and epidemiologic study types.

The evidence-based mapping strategies using SSB and T2D as an example facilitated the logical grouping of studies on key design features and suggested subgroups of studies appropriate for statistical summation via meta-analysis. For example, we found considerable variability in the definition and methods of collection of the exposure variable (sweetened beverages). Most notable is the inclusion of artificially-sweetened beverages in the definition in 3 of 11 cohorts. Consequently two broad research questions are addressed by this series of selected studies: T2D risk associated with intake of 1) SSB only and 2) any soft drink (artificially or sugar-sweetened). Diversity across studies in the definition of the exposure variable may be due to a combination of factors, including availability of data from the dietary assessment tool, the definition used by the study investigators, or the level of detail provided in the publication. Improving the interpretability of meta-analyses will require investigators of primary studies, in as much as possible, to define variables, conduct analyses, and report findings with an eye towards how their results may be compared to or possibly combined with other studies in the future.

The systematic approach described herein culminated in a prototype for a table that can be employed widely for reporting the extent and multi-dimensional nature of design heterogeneity across eligible studies in a meta-analysis. This table is recommended in addition to the classic table 1 in a systematic review (which usually describes studies individually). A standard table summarizing design heterogeneity across all selected studies will bring to the fore many elements necessary for interpreting the pooled risk estimate from a meta-analysis. One of many examples from our assessment of cohorts of SSB and T2D is 7 of 11 studies measured beverage intake only once at baseline, each using a different diet questionnaire and following participants for T2D from 7 to 16 years. The etiologically relevant time period for most chronic diseases, including T2D, is most often not known, and a one-time measurement of dietary intake may not capture intake in the relevant time frame. This is a fundamental consideration when interpreting results of chronic disease studies, including meta-analysis of these data.

To our knowledge, this may be the first detailed report of diversity of statistical modeling approaches among observational studies selected for a meta-analysis. A common practice for reporting modeling strategies in meta-analyses of observational studies is to provide a list of included covariates by study. We suggest summarizing the following across selected studies: the number of multivariable models presented, the number of covariates adjusted for in multivariable models, and the fluctuation in the fully adjusted risk estimate relative to the age-adjusted or most minimally adjusted model. As an example, it may add confidence about the results of a meta-analysis combining models that all adjust for the same 5 variables and with little fluctuation in the most fully adjusted risk estimate compared to the age or most minimally adjusted model. In contrast, cohorts of SSB and T2D reported up to 8 multivariable models adjusting for between 11 and 17 covariates. The results revealed a 2-95% reduction in risk of T2D associated with sweetened beverage consumption in fully adjusted relative to minimally/age- adjusted models (Table 2). The latter finding was most pronounced among the eight studies of SSB only, where adjustment for between 11 to 17 covariates resulted in a 46 to 95% reduction in the SSB-T2D risk estimate (Table 2). In other words, in many studies adjustment for covariates explained half to all of the association between SSB and T2D and should be considered when analyzing and interpreting a meta-analysis of the data.

Selection of statistical models by the study investigators from the primary publication and by a review investigator for a meta-analysis also influences the final outcome of a pooled analysis of observational studies. This particular bias, called selective analysis reporting, has recently been discussed as a major concern for meta-analysis of nonrandomized studies, but also applies to observational etiologic investigations [57]. Covariate selection and modeling strategies require careful consideration in the final interpretation of a pooled analysis of SSB and T2D; a single estimate for a pooled risk may be an oversimplification of complex data. Evidence maps can help facilitate the selection of additional models for sensitivity analyses in a meta-analysis.

Heterogeneity of studies can be a reason not to perform a meta-analysis. For example, a systematic review of whole grain foods and T2D that had intended to complete a meta-analysis concluded a qualitative synthesis was more appropriate for the data [58]. Other investigators have determined that a meta-analysis of this topic was informative [59,60]. A tool (such as the proposed summary table) that clearly displays design heterogeneity may be helpful in weighing both sides of this type of debate.

Systematic reviews and meta-analyses are well accepted research synthesis methods that serve to inform researchers, policy makers and, increasingly, the public of the potential causes of disease and the extent to which disease (or preventive) interventions are effective. The efficiency of these efforts depends largely on the quality of data from primary studies and a clear assessment of the extent to which that data can be combined.

## Conclusions

We illustrate a framework employing evidenced-based mapping to organize, evaluate and document design heterogeneity. This exercise culminated in a recommendation for a standardized table format that clearly summarizes design heterogeneity of eligible studies, with the goal of informing a protocol for meta-analysis and subsequently facilitating interpretation of summary risk estimates after quantitative synthesis. We recommend expanding the practice of meta-analysis of cohort studies to include a standard table that summarizes design heterogeneity. Addition of this table to reporting of meta-analyses provides the reader with more evidence to interpret the summary risk estimates.

## Abbreviations

ARIC: Atherosclerosis Risk in Community Study; BMI: body mass index; BWHS: Black Women’s Health Study; EPIC: European Investigation of Cancer; MESA: Multi-Ethnic Study of Atherosclerosis; NHS: Nurse’s Health Study; PICO: participants, exposure/intervention, comparator, and outcome; SD: soft drinks; SSB: sugar-sweetened beverages; T2D: type 2 diabetes.

## Competing interests

This methodological study was supported by The Coca-Cola Company. By contractual agreement, decisions regarding the content of the manuscript − including the design, analysis and interpretation − rest solely with the authors.

## Author’s information

MDA and DLW are independent consultants. MDA works for (and owns) EpiContext. DLW works for (and owns) DLW Consulting Services. CLF is a faculty member at George Mason University. This work was undertaken with the contractual understanding that the funder had no control over the design, analysis, and interpretation of the study and its results.

## Authors’ contributions

All authors (MDA, DLW, CLF) contributed to the concept, design, interpretation of data and writing of the manuscript. MDA and CLF completed data abstraction and quality control. MDA created the evidence maps and tables. All authors (MDA, DLW, CLF) read and approved the final manuscript.
